# Autonomic evidence that avoidance matters in the mourning process: A prospective observational study in Japan

**DOI:** 10.1080/19585969.2025.2597058

**Published:** 2025-12-08

**Authors:** Takuya Yoshiike, Tomoki Yajima, Tomohiro Utsumi, Srishti Tripathi, Aoi Kawamura, Kentaro Nagao, Kentaro Matsui, Yoko Matsuda, Mitsunari Abe, Masaya Ito, Satomi Nakajima, Kenichi Kuriyama

**Affiliations:** ^a^ Department of Sleep-Wake Disorders, National Institute of Mental Health, National Center of Neurology and Psychiatry, Tokyo, Japan; ^b^ Cognitive Behavioral Therapy and Research Institute, Musashino University, Tokyo, Japan; ^c^ Department of Advanced Neuroimaging, Integrative Brain Imaging Center, National Center of Neurology and Psychiatry, Tokyo, Japan; ^d^ Department of Research and Development, National Center for Cognitive Behavior Therapy and Research, National Center of Neurology and Psychiatry, Tokyo, Japan; ^e^ Faculty of Human Sciences, Musashino University, Tokyo, Japan

**Keywords:** Avoidance, bereavement, heart rate variability, prolonged grief, autonomic nervous system

## Abstract

**Introduction:**

Evidence provides support for the therapeutic benefits of targeting avoidance in prolonged grief. However, it is not clear whether avoidance interferes with mourning through altered resilience to stress, as measured by heart rate variability (HRV).

**Methods:**

Thirty-five adults (30 female; mean age: 39.2 years), who had been bereaved for more than one year, participated in this prospective, observational study. At each of the initial assessments and up to six-month follow-ups, grief symptoms were assessed using the Complicated Grief Questionnaire, and a resting electrocardiogram was recorded to extract the high-frequency component of HRV (HF-HRV). To differentiate avoidance from grief itself, principal component analysis was used.

**Results:**

A nonlinear cross-sectional relationship was observed between avoidance and HF-HRV (coefficient = 0.29, *p* = .003); the lower the avoidance, the lower the HF-HRV in the low avoidance group. Grief improved only in the low avoidance group longitudinally. The observed relationship between increased HF-HRV and decreased grief was modified by the avoidance group, such that the low-avoidance group drove this association (estimate −0.53, 95% CI −0.86, −0.21, *p* = .001), while the high-avoidance group did not (estimate 0.44, 95% CI −0.32, 1.20, *p* = .26).

**Conclusion:**

Despite its palliative gain, avoidance relates to the maintenance of grief longitudinally through attenuated autonomic resilience to stress.

## Introduction

Evidence is mounting on the health outcomes of bereavement (Stroebe et al. 2007; Aiello et al. 2024). Prolonged grief disorder (PGD), which affects 10% of bereaved adults (Lundorff et al. 2017), is a major public health concern worldwide (Boelen and Smid 2017), and has recently been included in the International Classification of Diseases, 11th Revision (ICD-11) and Diagnostic and Statistical Manual of Mental Disorders, Fifth Edition, Text Revision (DSM-5-TR). However, standard prevention and treatment strategies for prolonged grief have yet to be established.

Despite the reported clinical benefits of exposure to the reality of loss (Bryant et al. 2014; Shear et al. 2016; Bryant et al. 2017), the mechanisms by which avoidance disturbs the mourning process are not well understood. Yet, it is clinically known that some bereaved individuals can even benefit from avoidance, particularly during acute grief (Shear et al. 2007). There are two issues that require further consideration. First, it is difficult to differentiate the avoidance of ‘the reality of loss’ from the avoidance of ‘traumatic aspects of loss’. No previous biological studies have focused on the former aspect of avoidance (Freed et al. 2009; Schneck et al. 2017). Second, although there are questionnaires that have been developed to assess avoidance behaviour in the context of grief, such as the Grief-Related Avoidance Questionnaire (GRAQ) (Shear et al. 2007), their total scores have been found to correlate with grief itself (Baker et al. 2016). Therefore, they would not provide a specific focus on avoidance that is separate from grief itself or trauma-related avoidance. This underscores the need for an alternative method of assessing this behaviour. One approach to overcoming these limitations is to pursue objective correlates of avoidance behaviour that is more specifically quantified.

Autonomic dysfunction may be a mechanism tying bereavement to poor health outcomes, including prolonged grief (Stroebe et al. 2007; O’Connor 2019). The stress theory’s basic assumption is that stressful life events play an important role in precipitating the onset of various types of illnesses, through the brain’s influence on physiological responses to psychosocial stress. Evidence suggests that bereavement may increase susceptibility to infectious diseases, cancer, cardiovascular diseases, and psychiatric disorders by impairing the autonomic nervous system (ANS) that regulates immune, endocrine, cardiac, and mental functions (Parkes et al. 1969; Gerra et al. 2003; Stroebe et al. 2007; Mostofsky et al. 2012; Wei et al. 2022). Heart rate variability (HRV), a non-invasive method to evaluate the modulation of the ANS on the sinoatrial node, can not only serve as an indicator of cardiac function, but also reflect the central modulation capacity to cope with different types of stressors, including bereavement (Olivieri et al. 2024). There is a consensus that HRV reflects one’s psychophysiological resilience, with higher HRV indicating greater resilience (An et al. 2020; Perna et al. 2020; Tupitsa et al. 2023). This is relevant to understanding the clinical outcomes of bereavement.

We first assumed that bereaved people would eventually experience a reduction in grief, even after a significant amount of time had passed since their loss, with or without intervention. This aligns with recent findings from population-based studies showing that 75–93% of bereaved individuals experienced a decrease in grief symptoms over two to three years (Nielsen et al. 2019; Djelantik et al. 2022). We also assumed that the maladaptive aspects of avoidance strategies would emerge later, after a loss. This assumption was based on the idea that avoidance strategies can be used adaptively during the initial period following a loss (Shear et al. 2007; Smith et al. 2024). Conversely, confrontation with the reality of loss—the essence of adaptive grieving—could occur at any time within confrontation-avoidance dynamics (Stroebe & Schut 1999) and might be triggered by some cognitive processes, such as realising one’s symptoms of grief may deviate from the norm. Given the clinical relevance of addressing avoidance and its biological correlates in coping with grief, the present study aimed to determine the association between avoidance and HRV in the reduction of grief on a longitudinal basis. It is hypothesised that avoidance would help maintain HRV, but postpone the mourning process.

## Methods

### Eligibility criteria

All study procedures were approved by the Ethics Committee of the National Centre of Neurology and Psychiatry before recruitment. All participants provided written informed consent and were compensated for their participation. We recruited participants who were still experiencing symptoms of grief at least one year after their loss. An experienced psychiatrist confirmed the eligibility criteria through a clinical psychiatric interview and completed the Mini-International Neuropsychiatric Interview (Version 7.0.2) (Sheehan et al. 1998). Eligible participants were at least 20 years of age and had been bereaved of a close person (e.g., a child, spouse, parent, sibling, grandparent, or close friend) for more than 12 months before enrolment. Participants were recruited between May 2022 and January 2025 using various methods, such as the study website, social media, or local advertisements. The exclusion criteria were current psychiatric disorders, except for PGD, depressive disorders, post-traumatic stress disorder (PTSD), or anxiety disorders, based on the DSM-5-TR; substance use disorder in the past six months; severe suicidality in the past six months; cognitive impairment indicated by a score < 24 on the Mini-Mental State Examination; neurological disorders; pregnancy; and a medical condition that could interfere with the study or the interpretation of its results (e.g., a current diagnosis of cardiovascular disease). All participants were Japanese speakers, ensuring their understanding of the questionnaire. A total of 41 bereaved adults were assessed for eligibility, of which six were excluded because they met at least one exclusion criterion (*n* = 1) or declined to participate (*n* = 5), resulting in a final sample of 35 bereaved adults, who completed an initial assessment. Of these, 26 (74.3%) participants agreed with and completed a follow-up assessment. This study adhered to the Declaration of Helsinki.

### Study design and procedure

This was a prospective observational study. Participants underwent the initial assessment (T1) and the follow-up assessment 3–6 months after T1 (T2). At each assessment, participants completed questionnaires at home within one week before the electrocardiogram (ECG) recording that each participant underwent in a laboratory. At T1, the experienced psychiatrist made a diagnosis of PGD based on the DSM-5-TR criteria. In both assessments at T1 and T2, the Complicated Grief Questionnaire (CGQ) was used to determine the frequency of grief symptoms over the past month (Cozza et al. 2016). Although the CGQ is a self-report questionnaire that has yet to be validated in the literature, its scale was derived from the Structured Clinical Interview for Complicated Grief (Bui et al. 2015), a validated instrument that has been used to compare the diagnosis of DSM-5 persistent complex bereavement disorder, prolonged grief disorder, and complicated grief. The CGQ comprises 26 items, each of which is rated on a five-point Likert scale ranging from 0 (never) to 4 (very often). Total score range is 0–104, with higher scores indicating greater grief symptoms. The CGQ includes ten symptoms and two additional items related to distress and impairment that can be used to generate a DSM-5-TR criteria-based PGD diagnosis. For the cohort in the present study, Cronbach’s α for total score was .95. The Inventory of Complicated Grief (ICG) was also used for comparison with existing literature. Other questionnaires included the Grief-Related Avoidance Questionnaire (GRAQ) (Shear et al. 2007), Typical Belief Questionnaire (TBQ) (Skritskaya et al. 2017), which measures maladaptive cognitions in grief, Patient Health Questionnaire-9 (PHQ-9) for depressive symptoms (Muramatsu et al. 2007), PTSD Check List for DSM-5 (PCL-5) for PTSD symptoms (Ito et al. 2019), and EuroQol 5 dimensions 5-level (EQ-5D-5L) for participants’ quality of life (Brooks et al. 2003). After the ECG recording, the psychiatrist rated global illness severity using the Clinical Global Impression – Severity Scale (CGI-S) at T1 and T2. At T1, participants provided information on bereavement and health status, as well as sociodemographic information: when and how the loss occurred, age, sex, alcohol consumption, smoking status, body mass index (BMI) based on self-report, physical conditions, and psychiatric medications. Information regarding the use of bereavement support was collected at T1 and T2.

### Autonomic outcome

High-frequency (HF) component of HRV (HF-HRV, [ms^2^]) is assumed to be vagally mediated (Olivieri et al. 2024) and reflect one’s psychophysiological resilience (An et al. 2020; Perna et al. 2020; Tupitsa et al. 2023). In the cross-sectional analyses, HF-HRV was the primary outcome measure. Relatively high HRV is advantageous for both physiological and psychological health (Olivieri et al. 2024). At T1 and T2, a resting-state ECG was recorded with the participant in the supine position during a 10-min resting phase. The first half of the period was for acclimatisation to the recording environment, while the second half was used for analysis. On each occasion, the recording was conducted in the afternoon (between 15:30 and 16:30), given the diurnal variation in HRV. A Hexoskin Smart Shirt (Carre Technologies Inc., Montreal, Canada) with three built-in sensors (two beneath the chest and one on the right side of the abdomen) was used. The validity and reliability of this device have been demonstrated for determining HRV (Villar et al. 2015). The signal was sampled at 256 Hz, and the low-pass filter frequency was set to 35 Hz. The R-R intervals were derived using BIMUTAS-Video for the ECG (Kissei Comtec, Nagano, Japan). Irregularities in the R-R interval plots were visually detected in the raw electrocardiographic signal, and manually edited according to a well-established protocol to correct artefacts and abnormal beats (Berntson et al. 1997). HF-HRV, defined as the power between 0.15 and 0.4 Hz, was extracted using the autoregressive model as a frequency-domain-based index. Natural log transformation was applied to the HF-HRV to better approximate a normal distribution.

### Clinical predictor and outcome

In the cross-sectional analyses, severities of grief and avoidance were used as predictors. In the longitudinal analyses, grief severity was the primary outcome measure, whereas the change in CGI-S was a secondary outcome measure. Given our previous approach to differentiate between grief itself and avoidance, principal component analysis (PCA) was used, and the number of dimensions of the grief symptoms measured by the CGQ was reduced, assuming that the two grief presentations may represent biologically distinct constructs (Yoshiike et al. 2023). Individual CGQ item scores for the entire 61 observations over T1 (*n* = 35) and T2 (*n* = 26) were used for PCA. The significant components were extracted using v-fold cross-validation, while striking a balance between model complexity and the ability to accurately predict the data in the presence of eigenvalues > 1.

### Data analysis

Participant characteristics at T1 were summarised using mean (SD) and N (%), and compared between the low and high-avoidance groups, which were split by the median of avoidance component scores. Mann-Whitney U tests or Chi-square tests were used, where appropriate. Bivariate correlations of the grief and avoidance components with related grief and avoidance measures at T1 were tested using Spearman’s rank correlation coefficients.

#### Cross-sectional analyses

Using cross-sectional analyses, we tested whether levels of grief and avoidance were associated with HF-HRV levels. Considering its interpretability and generalisability, first, a linear regression was run to examine whether and how the grief and avoidance components were cross-sectionally related to HF-HRV. When a linear association was not supported, a nonlinear association was explored, given a possible optimal range in which avoidance would help maintain HRV against stress. A generalised linear model (GLM) with an identity link function and a generalised additive model (GAM) were used to test linear and nonlinear associations, respectively. In both cases, the first (grief) and second (avoidance) principal component scores were included as predictors.

Independent associations between grief or avoidance level and HF-HRV were tested in separate models adjusted for potential confounders. In addition to unadjusted and age/sex adjusted models, we ran three multivariable-adjusted models: Model 1 adjusted for bereavement factors, including years since loss, violent or sudden death, and child or spouse loss; Model 2 adjusted for lifestyle and physical factors, including alcohol consumption, smoking status, BMI, physical conditions; and Model 3 adjusted for psychiatric factors, including psychiatric medications, depressive symptoms, and PTSD symptoms. A slope analysis was conducted to examine whether the avoidance group modified the association between avoidance level and HF-HRV at T1. The same slope analysis was completed for avoidance level and HF-HRV at T2 to examine whether the association at T1 remained unchanged over time. Further, a preliminary slope analysis was conducted to test whether both the avoidance group and PGD diagnosis modified the association between avoidance and HF-HRV at T1.

#### Longitudinal analyses

In the longitudinal analyses, we tested whether an increase in HF-HRV led to a reduction of the grief level over time. The longitudinal analyses were conducted for complete cases (*n* = 26; low avoidance: *n* = 13; high avoidance: *n* = 13). Repeated-measures analysis of variance was used to examine whether changes in grief and avoidance levels (or changes in CGI-S) over time differed by avoidance group, followed by Fisher’s protected least significant difference tests. GLM was used to test whether a change in HF-HRV would predict a change in grief level (or change in CGI-S).

Independent associations of change in HF-HRV with change in grief level (or change in CGI-S) were tested in separate models. In addition to unadjusted and age/sex adjusted models, we ran two multivariable-adjusted models: Model 1 adjusted for bereavement factors, including years since loss, violent or sudden death, child or spouse loss; and Model 2 adjusted for intervention factors, including use of psychiatric medications, and use of bereavement support. A slope analysis was conducted to assess whether the avoidance group determined at T1 modified the association between change in HF-HRV and change in grief level (or change in CGI-S). Further, a preliminary slope analysis was conducted to test whether both the avoidance group determined at T1 and PGD diagnosis modified the association between change in HF-HRV and change in grief level (or change in CGI-S).

Statistical analyses were conducted using the StatSoft Statistica software (version 12.0; StatSoft Inc., Tulsa, OK, USA), and a P-value < .05 was considered significant.

## Results

### Participant characteristics

At T1, the participants exhibited considerable grief severity on average, and 15 participants (42.9%) met the diagnostic criteria for PGD (Table 1). Compared with the low-avoidance group, the high-avoidance group had increased psychopathology in terms of pre-loss psychiatric history, maladaptive cognition in grief, grief-related avoidance, PTSD symptoms, and depressive symptoms. A total of 14 participants received some form of bereavement support, either partially or fully, during the study period. This support included grief-focused psychotherapy (*n* = 11) and general grief care at a psychiatric clinic (*n* = 3).

**Table 1.** Baseline characteristics by avoidance group.

Characteristics	All participants (*n* = 35)	Low avoidance (*n* = 18)	High avoidance (*n* = 17)	Z / χ^2^	P
Age, mean (SD), years	39.2 (11.5)	37.6 (8.8)	43.3 (13.6)	−1.17	.24
Sex, n (%)				1.91	.17
Female	30 (85.7)	14 (77.8)	16 (94.1)		
Male	5 (14.3)	4 (22.2)	1 (5.9)		
Education, mean (SD), years	15.9 (2.4)	16.0 (2.5)	15.8 (2.4)	0.23	.82
Time since loss, median (IQR), years	2.8 (1.6–6.8)	2.5 (1.6–5.6)	3.6 (1.9–14.0)	−1.06	.29
Age of person who died, mean (SD), years	54.7 (25.3)	53.4 (29.8)	56.2 (20.4)	−0.23	.82
Loss of a child or spouse, n (%)	13 (37.1)	8 (44.4)	5 (29.4)	0.85	.36
Violent or sudden death, n (%)	16 (48.4)	8 (44.4)	8 (47.6)	0.02	.88
Current smokers, n (%)	2 (5.7)	0 (0)	2 (11.8)	2.25	.13
Regular alcohol drinking, n (%)	9 (29.0)	6 (33.3)	3 (17.7)	1.13	.29
BMI, mean (SD), kg/m^2^	21.5 (4.6)	21.1 (3.2)	21.9 (5.9)	0.76	.45
Physical condition, n (%)	7 (22.6)	4 (23.5)	3 (21.4)	0.11	.74
Pre-loss psychiatric history, n (%)	16 (45.7)	5 (27.8)	11 (64.7)	4.80	**.028**
Psychiatric medication, n (%)	13 (37.1)	6 (33.3)	7 (41.2)	0.23	.63
Bereavement support, n (%)	14 (40.0)	9 (50.0)	5 (29.4)	1.54	.21
CGQ, mean (SD)	42.3 (24.2)	38.3 (27.2)	41.7 (19.4)	−1.45	.15
ICG, mean (SD)	29.7 (16.9)	27.9 (19.7)	29.2 (12.8)	−1.20	.23
TBQ, mean (SD)	41.4 (22.5)	32.4 (25.0)	50.9 (15.1)	−2.36	**.018**
GRAQ, mean (SD)	14.6 (12.8)	8.8 (10.8)	20.7 (12.2)	−2.89	**.004**
Prolonged Grief Disorder, n (%)	15 (42.9)	8 (44.4)	7 (41.2)	0.04	.85
PHQ-9, mean (SD)	9.9 (7.1)	7.1 (7.3)	12.8 (5.8)	−2.51	**.012**
PHQ-9 ≥ 10, n (%)	18 (51.4)	6 (33.3)	12 (70.6)	4.86	**.028**
PCL-5, mean (SD)	23.5 (18.1)	15.1 (16.6)	32.5 (15.4)	−3.07	**.002**
PCL-5 ≥ 33, n (%)	8 (22.9)	3 (16.7)	5 (29.4)	0.81	.37
EQ-5D-5L, mean (SD)	0.79 (0.20)	0.87 (0.18)	0.72 (0.19)	2.54	**.011**

Participants were divided into low and high avoidance groups according to the median of the avoidance component score. Bold font denotes significant results at *p* < .05.

Abbreviations: SD, standard deviation; IQR, interquartile range; BMI, body mass index; CGQ, Complicated Grief Questionnaire; ICG, Inventory of Complicated Grief; TBQ, Typical Belief Questionnaire; GRAQ, Grief-Related Avoidance Questionnaire; PHQ-9, Patient Health Questionnaire-9; PCL-5, PTSD-Check List for DSM-5; EQ-5D-5L, EuroQol 5 dimensions 5-level.

### Grief and avoidance components

A PCA identified two significant components of the CGQ, which explained 53.3% of the total variance (Q^2^ = 0.48). The first component involved most CGQ items and was interpreted as ‘grief’, while the second component mainly involved the avoidance CGQ item and was interpreted as ‘avoidance’, replicating our previous results from a different cohort (Table S1) (Yoshiike et al. 2023). While the grief component was strongly correlated with CGQ and ICG, the avoidance component was uncorrelated with these, but only weakly correlated with the PCL-5 trauma-related avoidance item scores (items 6 and 7) and the GRAQ total score (Figure S1). Moreover, repeated-measures analysis of variance showed that the association between time since loss and grief symptom level differed by component at the initial assessment (years since loss × component interaction: F_1,33_ = 13.39, _P_η^2^ = .289, *p* = .0009, Figure S2): Years since loss (natural log-transformed) was inversely associated with grief level (estimate −1.70, 95% CI −3.08, −0.32, *p* = .017) but positively associated with avoidance level (estimate 0.71, 95% CI 0.20, 1.21, *p* = .007).

### A nonlinear association between avoidance and HRV: The less avoidant, the lower HRV

**Figure 1.**
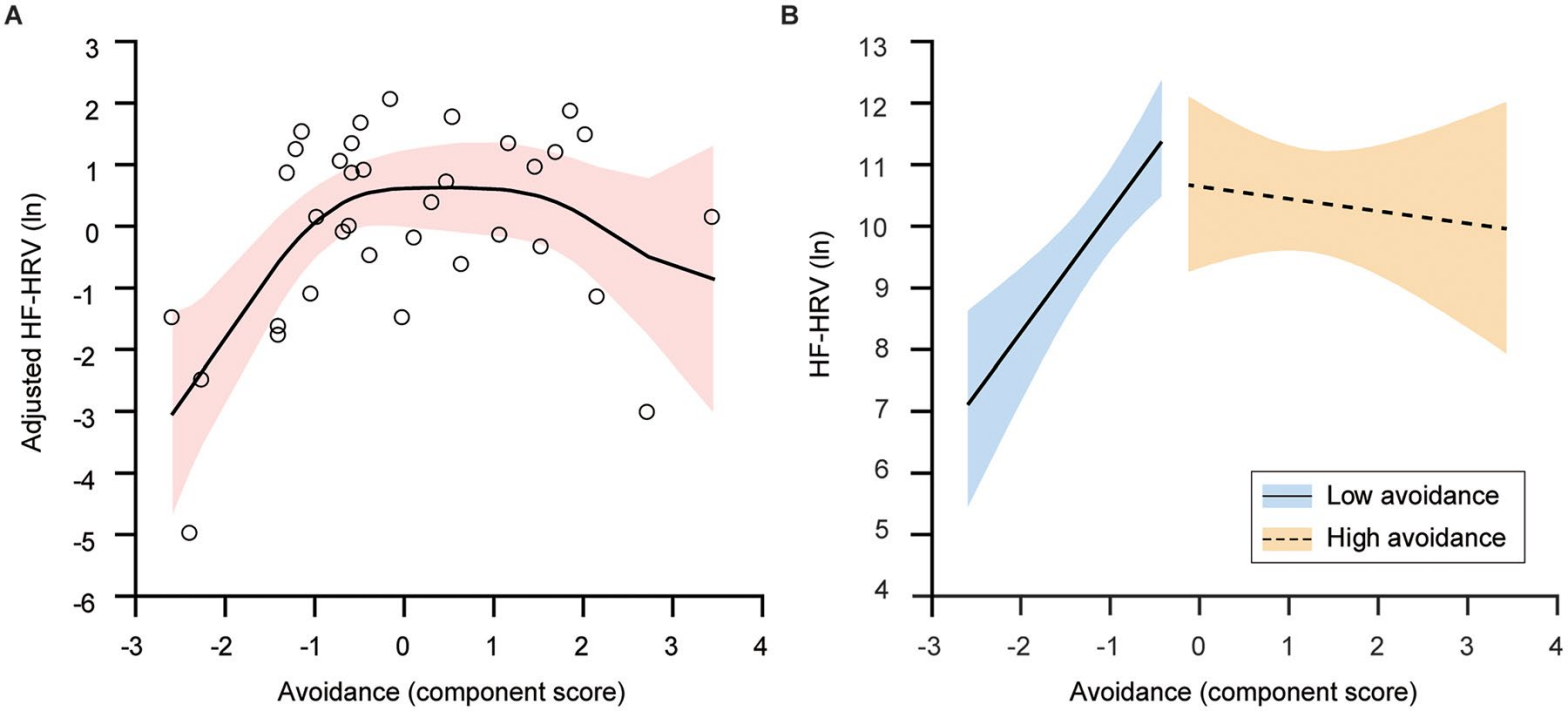
Cross-sectional association of avoidance with high-frequency heart rate variability (HF-HRV) at T1. (A) In a generalised additive model (GAM) analysis, the avoidance component score was nonlinearly associated with natural log-transformed HF-HRV. Open circles denote observed values. Values on the y-axis denote partial residuals of HF-HRV. A solid line and a red shaded area denote a spline line and 95% confidence intervals, respectively. (B) In a generalised linear model (GLM) slope analysis, avoidance component scores were positively associated with natural log-transformed HF-HRV in the low-avoidance group (*n* = 18), but not in the high-avoidance group (*n* = 17). Solid and dashed lines with blue and orange shaded areas denote regression lines and 95% confidence intervals for each avoidance group.

A linear association with HF-HRV was supported neither for grief (estimate −0.09, 95% confidence interval, CI −0.23, 0.04, *p* = .18) nor for avoidance (estimate 0.31, 95% CI −0.06, 0.68, *p* = .10). However, a nonlinear association was found between avoidance and HF-HRV, but not between grief and HF-HRV (avoidance level: effective degree of freedom [EDF] = 4.001, GAM coefficient [SE] = 0.29 [0.16], *p* = .003; grief level: EDF = 4.001, GAM coefficient [SE] = −0.08 [0.06], *p* = .14; Figure 1(A), Figure S3). The association between avoidance and HF-HRV was not affected by potential confounders (Table 2). This nonlinear association included at least two relationships: a positive relationship among participants with low avoidance, and a null relationship among those with high avoidance. Confirming the GAM results, a slope analysis in GLM revealed that the association between avoidance and HF-HRV was modified by the avoidance group, such that the low-avoidance group drove this association (estimate 2.05, 95% CI 1.11, 3.00, *p* < .0001), while the high-avoidance group did not (estimate −0.17, 95% CI −0.83, 0.49, *p* = .61; Figure 1(B)). The same patterns were observed with data at T2 (low avoidance: estimate 1.58, 95% CI 0.51, 2.66, *p* = .004; high avoidance: estimate −0.28, 95% CI −0.99, 0.44, *p* = .45). The positive association at T1 was further modified by PGD diagnosis, such that, the association remained significant in the low-avoidance with PGD group (estimate 1.72, 95% CI 0.65, 2.78, *p* = .002), but not in the low-avoidance without PGD group (estimate 1.31, 95% CI −0.60, 3.21, *p* = .18; Figure S4).

**Table 2.** Multivariable GAM analyses of HF-HRV by avoidance level at T1.

	EDF	Coefficient	SE	P
Unadjusted	4.001	0.29	0.16	.003
Age/sex adjusted	4.001	0.33	0.17	.024
Model 1	4.001	0.39	0.20	.005
Model 2	4.001	0.42	0.18	.020
Model 3	4.001	0.47	0.17	.011

Avoidance level was determined by principal component analysis of the Complicated Grief Questionnaire.

Model 1 adjusted for bereavement factors: years since loss, violent or sudden loss, and child or spouse loss.

Model 2 adjusted for lifestyle and physical factors: alcohol consumption, smoking status, body mass index, and physical conditions.

Model 3 adjusted for psychiatric factors: use of psychiatric medications, depressive symptoms, and post-traumatic stress symptoms.

Abbreviations: GAM, generalised additive model; HF-HRV, high-frequency heart rate variability; EDF, effective degree of freedom; SE, standard error.

### A longitudinal association between HRV and grief: The less avoidant, the more resilient

**Figure 2.**
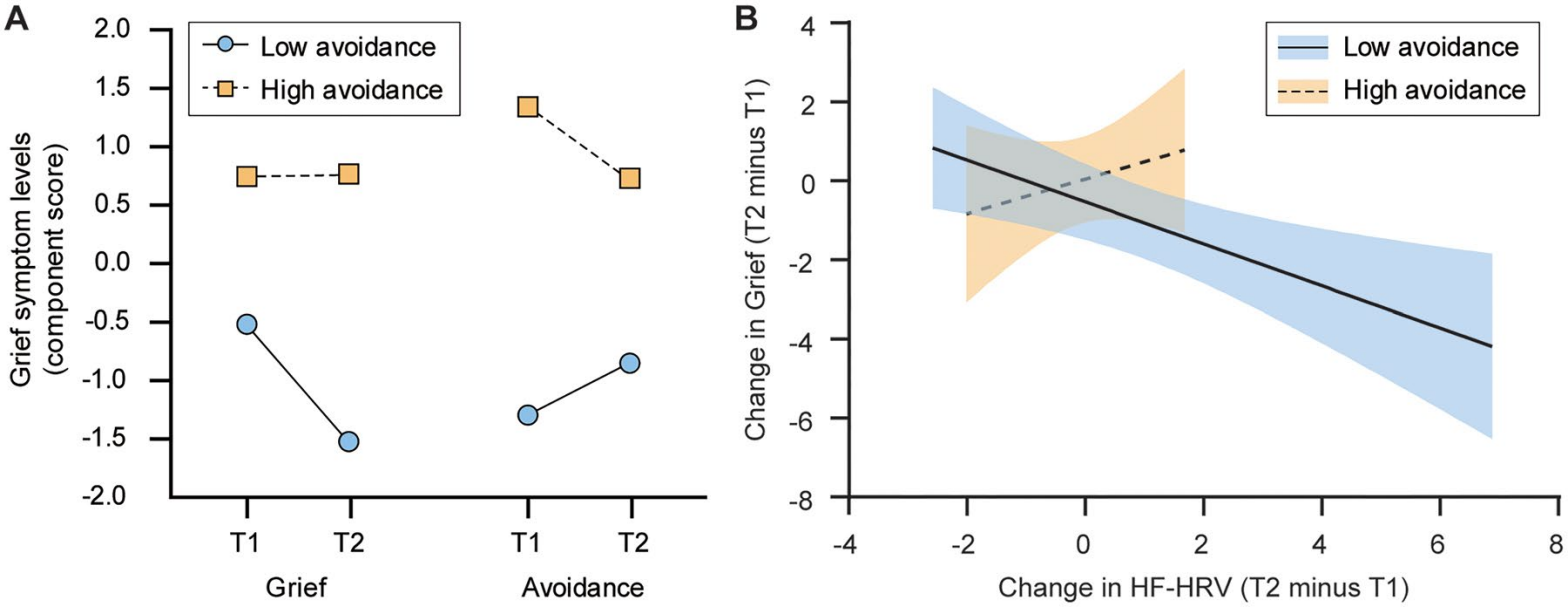
Longitudinal changes in grief symptoms and their association with changes in HF-HRV by avoidance group. (A) Grief component scores were reduced at T2 compared to T1 in the low-avoidance group (*n* = 13), whereas no significant change was observed in grief component scores in the high-avoidance group (*n* = 13). Avoidance component scores did not change significantly over time in either group. Regardless of time, grief and avoidance component scores were higher in the high-avoidance group than in the low-avoidance group. (B) An increase in natural log-transformed HF-HRV was associated with a reduction in grief component score in the low-avoidance group, but not in the high-avoidance group. A positive value on the x-axis denotes an increase in HF-HRV, whereas a negative value on the y-axis denotes a decrease in grief level from T1 to T2. Solid and dashed lines with blue and orange shaded areas denote regression lines and 95% confidence intervals for each avoidance group. Abbreviations: HF-HRV, high-frequency heart rate variability.

Participants were followed up after a median (interquartile range) of 103.5 (98–126) days. Changes in grief symptoms (i.e., grief and avoidance levels) differed by component and avoidance group (component × time × group interaction: F_1,24_ = 7.12, _P_η^2^ = .229, *p* = .013) such that grief, but not avoidance, decreased in the low, but not high-avoidance group (*p* = .017; Figure 2(A)). Grief symptoms were more severe in the high than in the low-avoidance group, regardless of time (main effect of group: F_1,24_ = 11.70, _P_η^2^ = .328, *p* = .002). A GLM revealed that the greater the increase in HF-HRV, the greater the reduction in grief level (estimate −0.42, 95% CI −0.74, −0.10, *p* =.011). The result was not affected by potential confounders (Table 3). In Model 1, which accounted for bereavement factors, years since loss was not significantly associated with change in grief level (estimate −0.024, 95% CI −0.155, 0.107, *p* = .72). This was also the case for change in CGI-S, a secondary outcome measure (estimate 0.006, 95% CI −0.045, 0.058, *p* = .80). The association between increased HF-HRV and decreased grief level was modified by the avoidance group such that the low-avoidance group drove this association (estimate −0.53, 95% CI −0.86, −0.21, *p* = .001), while the high-avoidance group did not (estimate 0.44, 95% CI −0.32, 1.20, *p* = .26; Figure 2(B)). The results were further modified by PGD diagnosis, with increased HF-HRV associated with decreased grief in the low-avoidance with PGD group (estimate −0.71, 95% CI −1.10, −0.31, *p* = .0005), whereas increased HF-HRV was associated with increased grief in the high-avoidance with PGD group (estimate 1.21, 95% CI 0.19, 2.24, *p* = .020; Figure S5). The results for CGI-S did not substantially differ from those for the primary clinical outcome measure (Table S2, Figures S5–S7).

**Table 3.** Multivariable GLM analyses of change in grief level by change in HF-HRV from T1 to T2.

				95% CI		
	Estimate	SE	Wald	LL	UL	P
Unadjusted	−0.421	0.165	6.50	−0.744	−0.097	.011
Age/sex adjusted	−0.377	0.171	4.84	−0.713	−0.041	.028
Model 1	−0.413	0.169	5.97	−0.745	−0.082	.015
Model 2	−0.465	0.153	9.30	−0.764	−0.166	.002

Grief level was determined by principal component analysis of the Complicated Grief Questionnaire, and its change over time was calculated by subtracting the grief component score at T1 from the grief component score at T2. Likewise, the change in HF-HRV over time was calculated by subtracting HF-HRV at T1 from HF-HRV at T2.

Model 1 adjusted for bereavement factors: years since loss, violent or sudden loss, and child or spouse loss.

Model 2 adjusted for intervention factors: use of psychiatric medications and use of bereavement support.

Abbreviations: GLM, generalised linear model; HF-HRV, high-frequency heart rate variability; SE, standard error; CI, confidence interval; LL, lower confidence interval limit; UL, upper confidence interval limit.

## Discussion

This prospective observational study revealed two aspects of avoidance in coping with grief: 1) avoidance as a palliative rescue of HRV, and 2) avoidance as a long-term hindrance of the mourning process. More specifically, bereaved adults with low avoidance had decreased HRV on cross-sectional analyses at the first time point, but were more likely to achieve a reduction in grief longitudinally, along with an increase in HRV. Conversely, high avoidance was not associated with HRV on cross-sectional analyses at the first time point; however, it also seemed to prevent recovery from grief in the long term.

To the best of our knowledge, this is the first study to provide evidence of an autonomic correlation between avoidance and its role in coping with bereavement. By extracting avoidance from grief symptoms, this study provided a glimpse of the possible underlying mechanisms allowing avoidance to act as both an autonomic defense response and a major obstacle to recovery from grief. This is not surprising, given that grief encompasses multiple ambivalent dimensions that might be biologically heterogeneous. The observed lower HRV in bereaved adults with PGD than in those without, aligns with previous observations in other stress-related conditions or situations (Buckley et al. 2012; Fagundes et al. 2018; Koch et al. 2019).

This study’s findings provide an opportunity to objectively understand the advantages and disadvantages of avoidance. Research has shown that exposure to loss reminders can increase systolic blood pressure (Palitsky et al. 2023) and reduce HRV (O’Connor et al. 2007), as can be observed with other types of stress, indicating a potential need for addressing the autonomic burden of confronting the loss throughout bereavement, even if confronting the loss has been known as an essential element for going through grief. In line with this perspective, it was observed that the less avoidant the participant, the lower the HRV on cross-sectional analyses at the first assessment. Nevertheless, the bereaved participants with low avoidance were more likely than those with high avoidance to achieve better clinical outcomes in terms of grief itself and global illness severity. This finding suggests that confronting the reality of loss (or reducing avoidance behaviour) eventually enhances autonomic resilience to psychosocial stress in prolonged grief. Despite this, more intervention trials are needed to test the hypothesis that exposure to loss promotes mourning across cultures, before we can make practical recommendations based on our current results. If this hypothesis is supported, we would also need to determine how bereaved individuals can achieve this outcome, whether with or without the assistance of a therapist or healthcare provider.

Conversely, avoidance of loss reminders may alleviate some of this autonomic burden. However, a dilemma may arise because it could potentially prolong the mourning process over the long term (Shear et al. 2007), as poor grief outcomes, as well as increased pre- and post-loss psychopathology, were observed in the high-avoidance group compared to the low-avoidance group. Therefore, future research will examine whether bereaved individuals with more avoidant characteristics requires special considerations for heightened vulnerability to stress in order to balance physical and mental health when confronting their loss. This assumes that avoidance may serve as a defense mechanism that aids the regulation of autonomic homeostasis.

Despite the limited ability to perform rigorous subgroup analyses due to small sample sizes in this study, the diagnosis of PGD, which was almost evenly distributed between the low and high-avoidance groups, appeared to play a role in amplifying the effects of the low or high avoidance groups. More specifically, it seemed that the low avoidance group with PGD had decreased HRV in the cross-sectional assessments. It also seemed that in the longitudinal assessments, the low avoidance group with PGD had decreased grief symptoms with increased HRV over time, while the high avoidance group with PGD had increased grief symptoms as HRV increased. Future well-designed and statistically powered studies can better assess the clinical relevance of different PGD phenotypes (e.g., less avoidant PGD and more avoidant PGD), and possible phenotype-specific approaches across psychiatric and public health perspectives.

### Strengths and limitations

This study’s strengths included a novel approach to quantify avoidance, a clear focus on prolonged grief, and a longitudinal study design. However, it had several limitations. The small sample size and additional subgroup analyses may inflate the risk of Type I and Type II errors in the interpretations of the study results; the subgroup analyses and overall robustness of the findings should be interpreted with caution. However, the consistency of the findings between the clinical outcome measures (i.e., grief component and CGI-S score) provides novel insight into the role of avoidance in the mourning process. Heterogeneity in characteristics of loss (e.g., kinship, situation) may also be considered a factor that limits generalisability. All participants were Japanese speakers, which is a strength in terms of cultural specificity but could also limit generalisability. Although preliminary and somewhat outdated, a previous study reported that Japanese widows in Tokyo more often tried to escape reminders of their deceased spouses than did London widows (55% vs. 18%), while also more often sensing their spouses’ presence (90% vs. 50%) (Yamamoto et al. 1969). These descriptions imply that loss-related avoidance strategies may be more impactful determinants of the mourning process in Japanese culture than in other cultures. Future studies may address how grief and avoidance are expressed differently across cultures, as well as which biological factors interact to drive or diminish these behavioural expressions.

Since we expected that bereaved individuals may need more time to recognise that their grief is abnormal before deciding to participate in a study, we did not set an upper limit on how long ago the loss occurred for participation in the study. However, a longer time period since the loss occurred may increase the likelihood of confounding factors affecting the outcomes. Despite rigorous adjustments for potential confounders, residual confounding cannot be ruled out, such as physical activity, sleep, changes in income and multiple losses, and thus, caution should be exercised when interpreting our findings. Moreover, the reliance on a single physiological marker (i.e., HF-HRV) and the absence of multimodal biomarkers (e.g., cortisol, inflammatory markers) may be considered under limitations. Although our results suggest an independent association between change in HF-HRV and grief outcomes, irrespective of interventions such as bereavement support and psychiatric medications in multivariable-adjusted models, future intervention trials are necessary to better determine the independent effect of avoidance on trajectories of prolonged grief. Our method of quantifying avoidance still relied on self-reporting, indicating the need to develop a direct, objective measure of this important behaviour. Further research is required to better understand the hindrances to each individual’s mourning process and help bereaved individuals prevent and overcome the modifiable medical consequences of bereavement.

## Supplementary Material

Supplementary_Material_R2_DCNS.docx

## Data Availability

The data underlying this article will be shared upon reasonable request from the corresponding authors.
