# Supplementary material for: Autonomic evidence that avoidance matters in the mourning process: A prospective observational study in Japan
Source: Dialogues Clin Neurosci. 2025 Dec 8;28(1):1–10. doi: 10.1080/19585969.2025.2597058 (PMC12687905; doi:10.1080/19585969.2025.2597058)
Supplement: Supplementary_Material_R2_DCNS.docx [file TDCN_A_2597058_SM0531.docx]

**Supplementary Material**

**Autonomic evidence that avoidance matters in the mourning process: A prospective observational study in Japan**

**List of Supplementary Material**

**Table S1.** Principal Components of the Complicated Grief Questionnaire.

**Table S2.** Multivariable GLM Analyses of Change in CGI-S by Change in HF-HRV from T1 to T2.

**Figure S1.** Cross-correlation matrix.

**Figure S2.** Associations between time since loss and grief symptom levels by component.

**Figure S3.** Cross-sectional association of grief with HF-HRV at T1.

**Figure S4.** Cross-sectional associations between avoidance and HF-HRV by avoidance group and PGD diagnosis at T1.

**Figure S5.** Longitudinal associations between change in HF-HRV and change in grief level by avoidance group and PGD diagnosis.

**Figure S6.** Longitudinal changes in CGI-S and their association with changes in HF-HRV by avoidance group.

**Figure S7**. Longitudinal associations between change in HF-HRV and change in CGI-S by avoidance group and PGD diagnosis.

**Table S1. Principal Components of the Complicated Grief Questionnaire**

| **Grief** | | |  | **Avoidance** | | |
| --- | --- | --- | --- | --- | --- | --- |
| *No.* | *Symptom* | *Loading* |  | *No.* | *Symptom* | *Loading* |
| 6. | Unfairness | 0.890 |  |  |  |  |
| 20. | Feeling that life is meaningless | 0.856 |  |  |  |  |
| 26. | Difficulty to plan for the future | 0.853 |  |  |  |  |
| 21. | Feeling you can’t manage | 0.846 |  |  |  |  |
| 3. | Preoccupation with the deceased | 0.842 |  |  |  |  |
| 5. | Loneliness | 0.820 |  |  |  |  |
| 10. | Feeling shocked | 0.809 |  |  |  |  |
| 2. | Emotional pain | 0.796 |  |  |  |  |
| 23. | Feeling that a part of you died | 0.793 |  |  |  |  |
| 22. | Confusion about your role in life | 0.790 |  |  |  |  |
| 7. | A desire to die | 0.780 |  |  |  |  |
| 12. | Bitterness or anger | 0.765 |  |  |  |  |
| 16. | Not doing things you used to do | 0.762 |  |  |  |  |
| 9. | Feelings of disbelief | 0.757 |  |  |  |  |
| 19. | Feeling alone | 0.752 |  | 15. | Avoidance of loss reminders | 0.727 |
| 18. | Feeling envious of others | 0.723 |  |  |  |  |
| 1. | Yearning for the deceased | 0.692 |  |  |  |  |
| 14. | Physical or emotional reactions | 0.684 |  |  |  |  |
| 17. | Difficulty trusting other people | 0.683 |  |  |  |  |
| 13. | Negative thoughts about yourself | 0.674 |  |  |  |  |
| 8. | Troubling thoughts | 0.661 |  |  |  |  |
| 11. | Difficulty having positive memories | 0.637 |  |  |  |  |
| 4. | Dreams of the dead | 0.563 |  |  |  |  |
| 25. | Wanting to sense the deceased | 0.530 |  |  |  |  |
| 15. | Avoidance of loss reminders | 0.382 |  |  |  |  |
|  |  |  |  | 8. | Troubling thoughts | 0.359 |
|  |  |  |  | 11. | Difficulty having positive memories | 0.336 |
|  |  |  |  | 12. | Bitterness or anger | 0.322 |
|  |  |  |  | 20. | Feeling that life is meaningless | -0.340 |
|  |  |  |  | 25. | Wanting to sense the deceased | -0.446 |
|  |  |  |  | 1. | Yearning for the deceased | -0.476 |
|  | Eigenvalue | 13.85 |  |  |  | 1.87 |
|  | % Variance Explained | 53.27 |  |  |  | 7.19 |

Symptoms were rearranged in descending order for each component according to the ladings of the symptoms.

**Table S2. Multivariable GLM Analyses of Change in CGI-S by Change in HF-HRV from T1 to T2**

|  |  |  |  | 95% CI | |  |
| --- | --- | --- | --- | --- | --- | --- |
|  | Estimate | SE | Wald | LL | UL | P |
| Unadjusted | -0.182 | 0.066 | 7.69 | -0.310 | -0.053 | .006 |
| Age/sex adjusted | -0.163 | 0.068 | 5.75 | -0.297 | -0.030 | .016 |
| Model 1 | -0.173 | 0.066 | 6.85 | -0.303 | -0.044 | .009 |
| Model 2 | -0.180 | 0.067 | 7.21 | -0.311 | -0.049 | .007 |

Change in global illness severity was calculated by subtracting the CGI-S score at T1 from the CGI-S score at T2. Change in HF-HRV over time was calculated by subtracting HF-HRV at T1 from HF-HRV at T2.

Model 1 adjusted for bereavement factors: years since loss, violent or sudden loss, and child or spouse loss.

Model 2 adjusted for intervention factors: use of psychiatric medications, and use of bereavement support.

Abbreviations: GLM, generalized linear model; CGI-S, Clinical Global Impression – Severity Scale; HF-HRV, high-frequency heart rate variability; SE, standard error; CI, confidence interval; LL, lower confidence interval limit; UL, upper confidence interval limit.


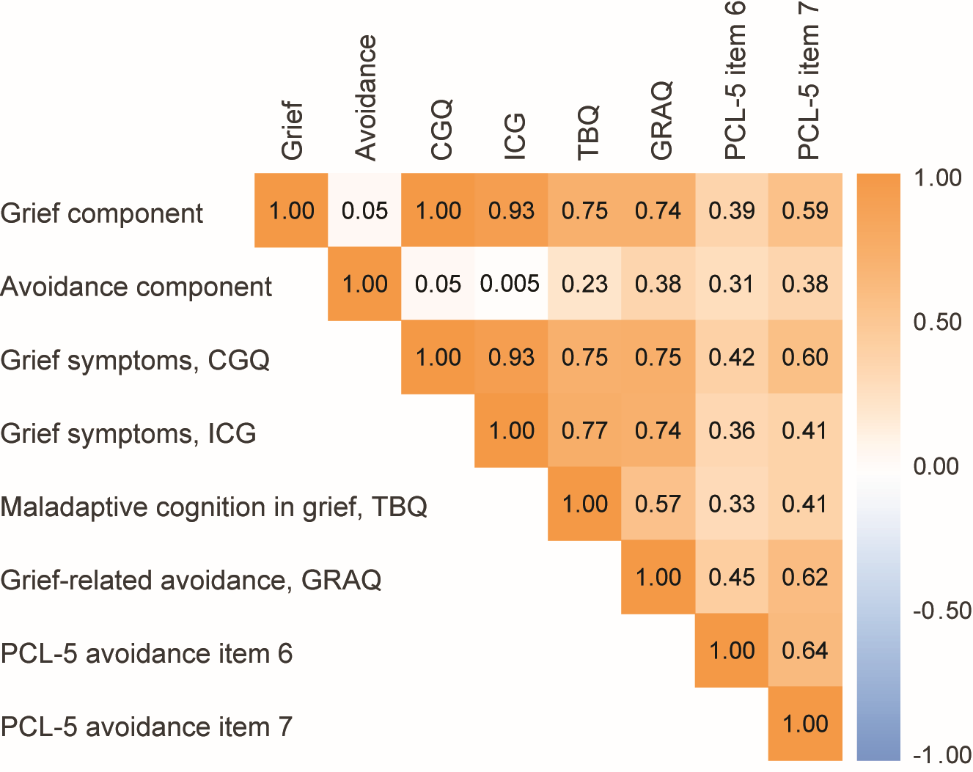


**Fig. S1.** Cross-correlation matrix. This matrix contains pairwise Spearman’s rank correlation coefficients between the two extracted principal components (i.e., grief and avoidance) and grief- and avoidance-related measures. Unlike the grief component, the avoidance component was not or only weakly associated with grief-related and other avoidance-related measures. Items 6 and 7 of the PCL-5 measured avoidance of memories, thoughts, or feelings related to the stressful experience and avoidance of external reminders of the stressful experience, respectively.

Abbreviations: CGQ, Complicated Grief Questionnaire; ICG, Inventory of Complicated Grief; TBQ, Typical Belief Questionnaire; GRAQ, Grief-Related Avoidance Questionnaire; PCL-5, PTSD Check List for DSM-5.


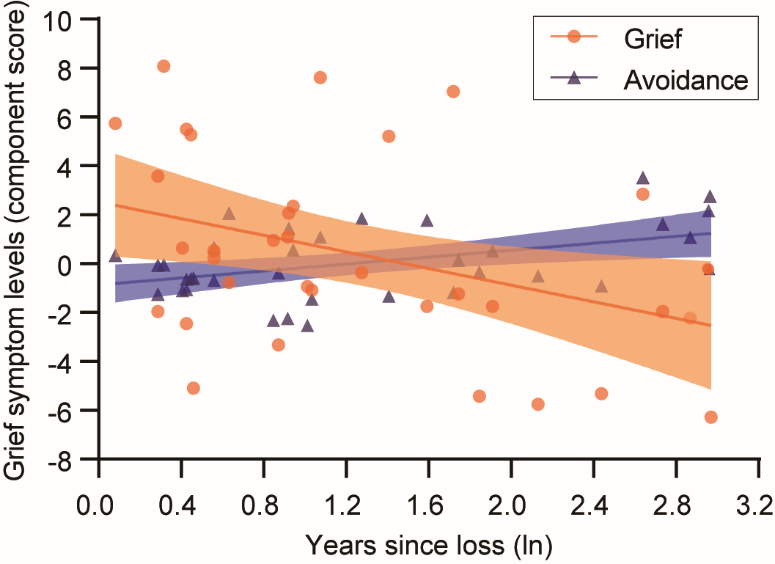


**Fig. S2.** Associations between time since loss and grief symptom levels by component. Solid lines and shaded areas denote regression lines and 95% confidence intervals for grief and avoidance components.

**
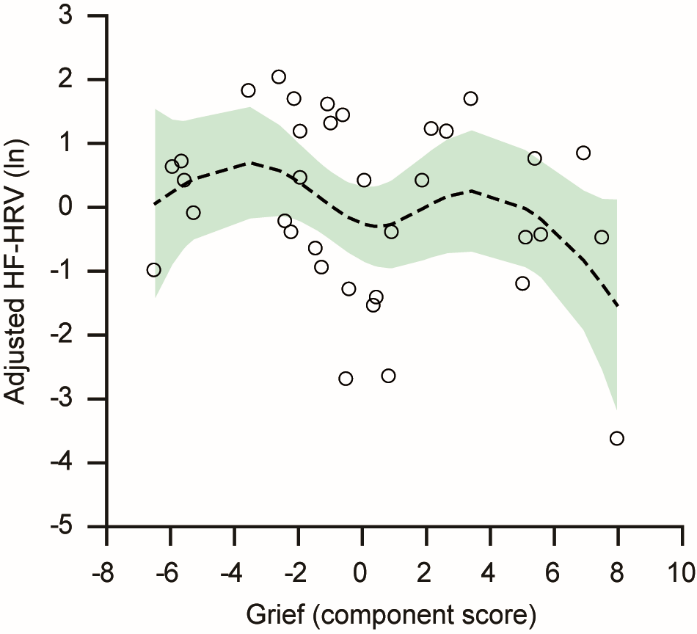
**

**Fig. S3.** Cross-sectional association of grief with HF-HRV at T1. In a GAM analysis, the grief component score was not significantly associated with natural log-transformed HF-HRV. Open circles denote observed values. Values on the y-axis denote partial residuals of HF-HRV. A dashed line and a green shaded area denote a spline line and 95% confidence intervals, respectively.

Abbreviations: HF-HRV, high-frequency heart rate variability; GAM, generalized additive model.


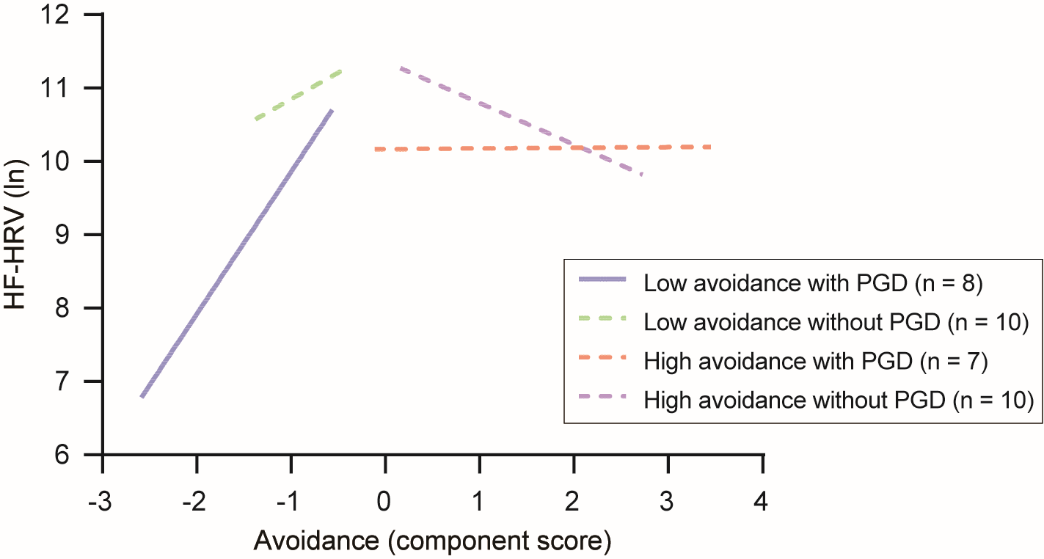


**Fig. S4.** Cross-sectional associations between avoidance and HF-HRV by avoidance group and PGD diagnosis at T1. PGD diagnosis modified the effect of the avoidance group on the association between avoidance component score and natural log-transformed HF-HRV, such that the association remained significant in the low-avoidance with PGD group, but not in the low-avoidance without PGD group. Solid and dashed lines denote regression lines for each classification.

Abbreviations: HF-HRV, high-frequency heart rate variability; PGD, prolonged grief disorder.

**
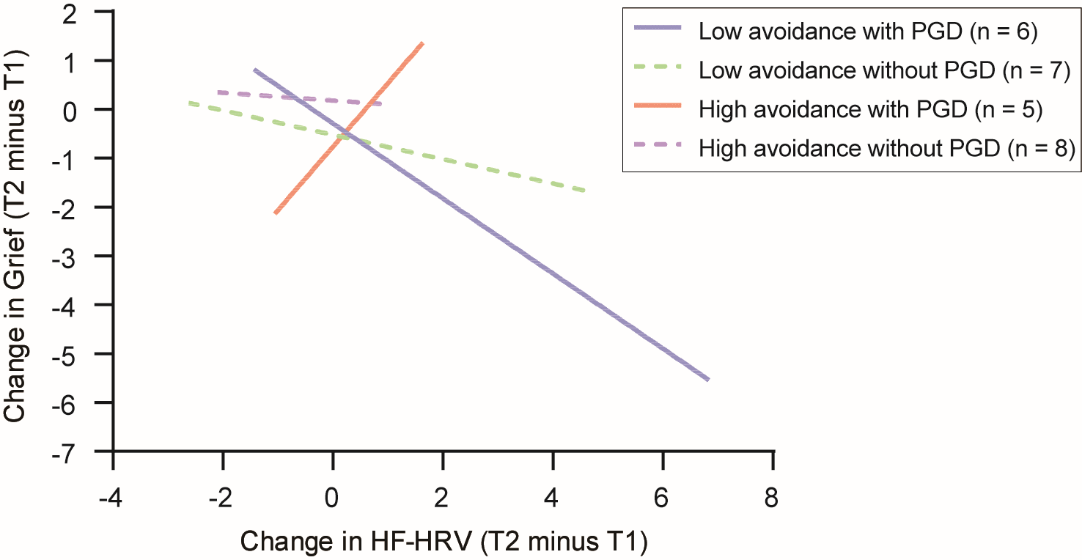
**

**Fig. S5.** Longitudinal associations between change in HF-HRV and change in grief level by avoidance group and PGD diagnosis. An increase in natural log-transformed HF-HRV was longitudinally associated with a decrease in the grief component score in the low-avoidance with PGD group, but was associated with an increase in the grief component score in the high-avoidance with PGD group. Solid and dashed lines denote regression lines for each classification.

Abbreviations: HF-HRV, high-frequency heart rate variability; PGD, prolonged grief disorder.


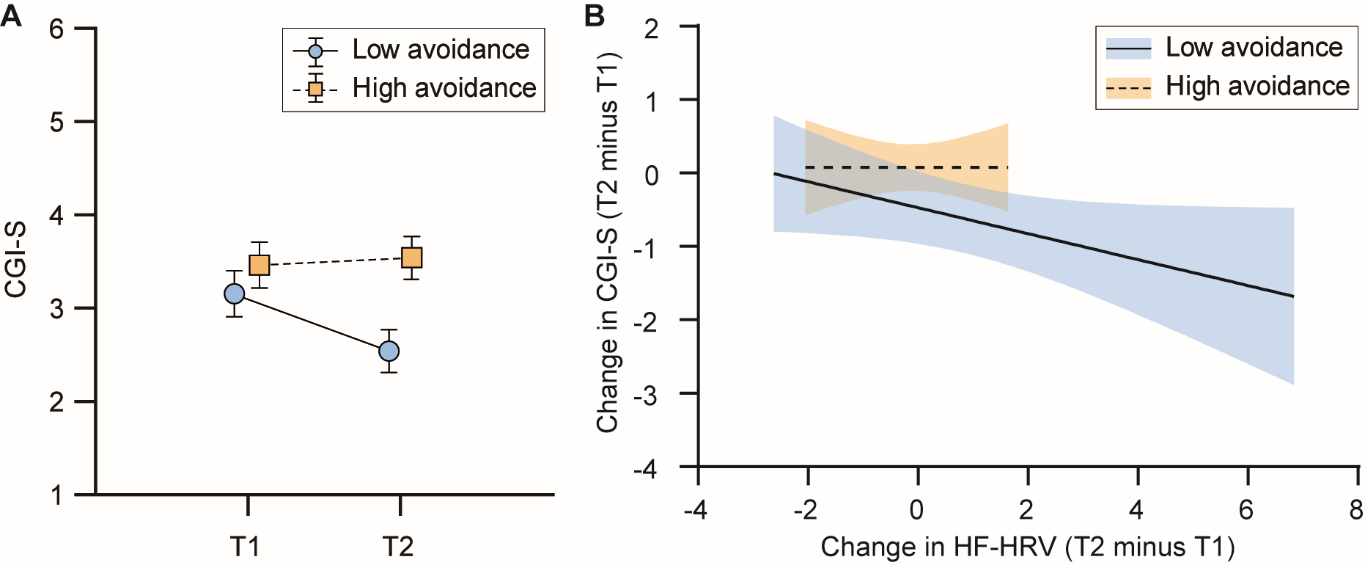


**Fig. S6.** Longitudinal changes in CGI-S and its association with changes in HF-HRV by avoidance group. (A) Change in CGI-S, a secondary outcome measure, also differed by avoidance group (time × group interaction: F_1,24_ = 6.23, _P_η^2^ = .206, P = .020), such that CGI-S decreased in the low- (n = 13) but not in the high-avoidance group (n = 13, P = .004). CGI-S was higher in the high than in the low-avoidance group, regardless of time (main effect of group: F_1,24_ = 4.54, _P_η^2^ = .159, P = .044). Error bars denote the standard error of the mean. (B) The association between increased natural log-transformed HF-HRV and decreased CGI-S was modified by the avoidance group, with only the low-avoidance group driving this association (low avoidance: estimate -0.18, 95% CI -0.31, -0.05, P = .007; high avoidance: estimate 0.0004, 95% CI -0.30, 0.30, P = 1.00). A positive value on the x-axis denotes an increase in HF-HRV, whereas a negative value on the y-axis denotes a decrease in CGI-S score from T1 to T2. Solid and dashed lines with blue and orange shaded areas denote regression lines and 95% confidence intervals for each avoidance group.

Abbreviations: CGI-S, Clinical Global Impression Severity Scale; HF-HRV, high-frequency heart rate variability.


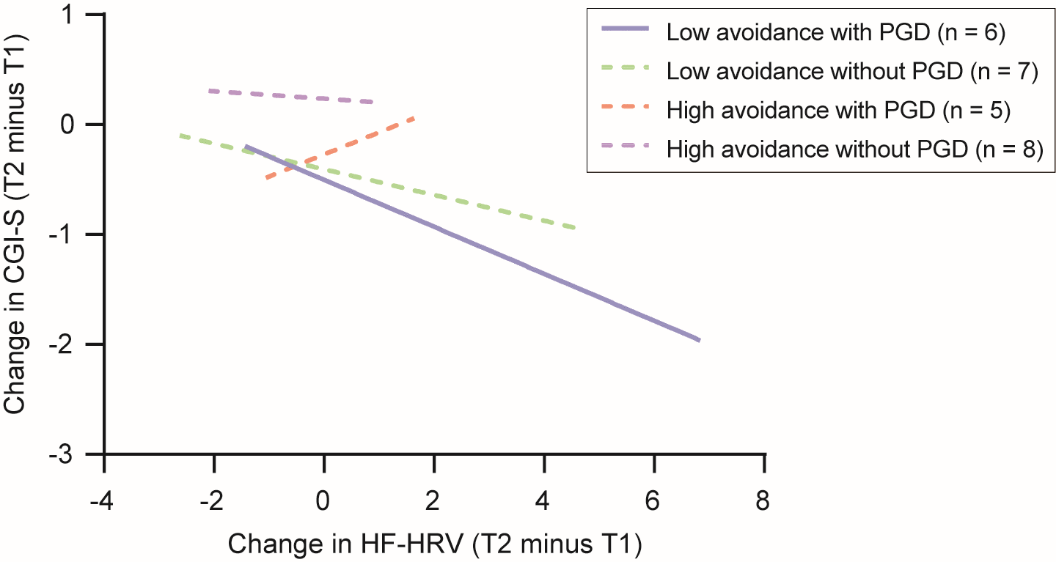


**Fig. S7**. Longitudinal associations between change in HF-HRV and change in CGI-S by avoidance group and PGD diagnosis. The association between increased natural log-transformed HF-HRV and decreased CGI-S was modified by avoidance group and PGD diagnosis, with only the low-avoidance with PGD group driving this association (estimate -0.19, 95% CI -0.36, -0.02, P = .025). Solid and dashed lines denote regression lines for each classification.

Abbreviations: HF-HRV, high-frequency heart rate variability; CGI-S, Clinical Global Impression Severity Scale; PGD, prolonged grief disorder.

.
